# Medical students on the COVID-19 frontline: a qualitative investigation of experiences of relief, stress, and mental health

**DOI:** 10.3389/fmed.2023.1249618

**Published:** 2023-11-09

**Authors:** Jennifer M. Klasen, Adisa Poljo, Rosita Sortino, Bryce J. M. Bogie, Zoe Schoenbaechler, Andrea Meienberg, Christian Nickel, Roland Bingisser, Kori A. LaDonna

**Affiliations:** ^1^Clarunis, Department of Visceral Surgery, University Centre for Gastrointestinal and Liver Diseases, University Hospital Basel, Basel, Switzerland; ^2^Faculty of Medicine, MD-PhD Program, University of Ottawa, Ottawa, ON, Canada; ^3^Faculty of Medicine, University of Basel, Basel, Switzerland; ^4^Department of Internal Medicine, Medical Outpatient Department, University Hospital Basel, Basel, Switzerland; ^5^Emergency Department, University Hospital, University of Basel, Basel, Switzerland; ^6^Department of Innovation in Medical Education and Department of Medicine, University of Ottawa, Ottawa, ON, Canada

**Keywords:** education, wellbeing, medical student, COVID-19, healthcare professional, qualitative study

## Abstract

**Objective:**

During the early stages of the COVID-19 pandemic, medical students were abruptly removed from clinical rotations and transitioned to virtual learning. This study investigates the impact of this shift on students’ wellbeing and preparedness for advanced training.

**Methods:**

Through qualitative research methods, including semi-structured interviews, the experiences of medical students working on the COVID-19 frontline were explored.

**Results:**

The comprehensive findings of the study shed light on the profound emotional journey that medical students embarked upon during the relentless public health crisis. Within the chaos and overwhelming demands of the pandemic, medical students discovered a profound sense of purpose and fulfillment in their contributions to the welfare of the community. Despite the personal sacrifices they had to make, such as long hours, limited social interactions, and potentially risking their own health, students reported feelings of relief and gratitude.

**Conclusion:**

Tailored support systems for medical students’ wellbeing are crucial for improving healthcare delivery during crises. Medical schools should adopt a holistic curriculum approach, integrating interdisciplinary learning and prioritizing student wellbeing. Recognizing the pandemic’s impact on students and implementing targeted support measures ensures resilience and contributes to an improved healthcare system.

## 1. Introduction

Medical students have experienced unprecedented disruptions in their personal and professional lives during the COVID-19 pandemic (1, 2). These disruptions included a shift to online learning, where many medical schools switched to remote teaching, impacting the quality of education and students’ ability to adapt (3). Clinical rotations and practical experiences, crucial for skills development, were often limited or canceled due to safety concerns (4). Students working in healthcare settings faced the risk of exposure to COVID-19, causing anxiety about their health (5). Licensing exams for medical professionals were frequently rescheduled or altered, adding stress for students preparing for these critical assessments. Financial hardships related to the pandemic affected students’ ability to cover tuition, living expenses, and other costs. Additionally, some students experienced delayed graduation as a result of disruptions in coursework and clinical training (6).

The COVID-19 pandemic has accelerated the already alarming rates of burnout and other mental health challenges experienced by clinicians and trainees alike (7). The current body of medical education research has extensively investigated the effects of the COVID-19 pandemic on healthcare workers and medical students independently, with many studies reporting negative outcomes for stress, fatigue, and somatic symptoms like insomnia, anxiety, anger, rumination, decreased concentration, depression and loss of energy (8, 9). In one survey, it was revealed that over half of the responding students (54.5%) indicated experiencing stress levels that spanned from moderate to extreme (10). According to a recent meta-analysis (11), the prevalence of depression symptoms among medical students during the COVID-19 pandemic was nearly twice that of the pre-pandemic estimates (12). Another systematic review and meta-analysis have unveiled a notably high prevalence of depression and anxiety among medical students. Specifically, the study found that the prevalence of depression stood at 37.9%, while anxiety was reported at 33.7%. These figures are notably higher when compared to the rates observed in the general population and among healthcare workers (13).

A limited number of medical students had the opportunity to work on the COVID-19 frontlines (14). This opportunity arose as medical schools and healthcare institutions across different countries recognized the heightened demand for extra support and manpower during this time of crisis. Consequently, medical education policies initiated various programs and initiatives to mobilize medical students to join healthcare teams. These initiatives aimed to bolster healthcare capacities and address the surge in demands brought about by the COVID-19 pandemic (14, 15). For instance, to mitigate the burden experienced by the local healthcare system, the University Hospital of Basel created an off-site Triage and Test Centre (TTC) which was located in the Preachers’ Church and staffed by an interprofessional team of nurses, physicians, and military service members to screen and evaluate patients with COVID-19-like symptoms (16, 17). Given their availability and eagerness to support the frontline care (18), medical students were recruited to work at the TTC voluntarily. Medical students were allowed to assist healthcare professionals by performing clinical assessments on numerous patients and conducting nasopharyngeal and oropharyngeal swabs. Additionally, they offered their support across various hospital departments, including different wards and the emergency department, to support the medical staff. The students were divided into two groups, with each group working for three consecutive days before rotating. To formalize their roles, each student received an official employment contract and comprehensive insurance coverage, ensuring that they were compensated for their valuable contributions. More details regarding this initiative can be found elsewhere (16). It’s important to note that the extent and nature of student involvement may have varied widely, depending on the specific circumstances and healthcare systems in different regions and at different times during the pandemic.

There is an urgent need for a deeper understanding of students’ wellbeing, including the factors that influence it and the impact of the pandemic on it. This is particularly crucial considering the pre-existing concerns regarding the wellbeing of medical students, which have been further intensified by the pandemic (19). Although existing research has touched on medical student wellbeing (20), the pandemic’s urgency and unprecedented circumstances demanded a dedicated study. While prior studies have provided valuable insights (21), there remains a knowledge gap in understanding the specific experiences and emotional journey of medical students who actively participated in crisis response teams. The rising rates of burnout and departures from medicine highlight the need to prioritize wellbeing among medical trainees. Understanding how medical students responded to the pandemic can inform strategies to enhance healthcare resilience during future crises (22, 23).

Since a recent study by Luong et al. suggested that educational adaptations and innovations implemented during the pandemic were perceived by some trainees as beneficial for fostering wellbeing (23), our purpose was to explore this finding with a unique cohort of learners. We’ve previously reported that medical students who volunteered at the TTC perceived the experience as meaningful in fostering their professional development and professional identity formation (16). Here, we delve into the experiences that impacted medical students’ personal health and wellbeing during the pandemic. By doing so, we aim to contribute to our understanding of trainees’ health and wellbeing and evaluate the outcome of pandemic-related adaptations. These findings have the potential to shed light on the challenges faced by medical students and assess the strategies put in place to protect their psychological wellbeing as the medical education landscape adapts to a new normal.

## 2. Materials and methods

Research ethics approval was obtained by the Ethics Commission of Northwest and Central Switzerland (EKNZ, Req-2021-00518).

### 2.1. Study design

This research followed a constructivist grounded theory (CGT) approach. CGT was chosen as the methodological approach for this qualitative study due to its ability to explore and generate theoretical insights that emerge from the data, allowing for a nuanced understanding of participants’ perspectives and experiences within the context under investigation. Given the rare circumstances of the study context and the understudied and theorized social processes such as learner wellbeing during COVID-19, this approach was also appropriate to investigate sensitive topics regarding participants’ experiences and perceptions during the pandemic.

### 2.2. Recruitment and participants

Participants were recruited via word-of-mouth through a TTC Chat Group, a private messaging group whose members comprised all medical students from the University of Basel who participated in the TTC. The interviewed participants suggested other possible interview candidates, therefore the recruitment strategy evolved into a snowball sampling approach, which allowed for the recruitment of less experienced students with little to no pre-pandemic bedside clinical training. Participants were sampled for both gender and level of training. Recruitment was stopped once theoretical sufficiency was achieved. This was operationalized as the point at which additional data failed to generate new knowledge. Hence, the data provided a sufficient description of the phenomena explored (24). Therefore, we concluded that the data collection after twenty-one semi-structured interviews. All interviews were audio-recorded, anonymized, and transcribed verbatim.

### 2.3. Study procedure and data collection

ZS interviewed all 21 participants between May and October 2020. The semi-structured interview guide was created by JK and ZS interviews were conducted in Swiss German, with selections translated into English for review by English-speaking team members. To ensure the interviews were conversational, we changed the interview language based on the participants as ZS interviewed them as a peer. The full interview guide can be find in the Supplementary material. During interviews, ZS explored participants’ experiences working on the COVID-19 frontlines, asking questions aimed at understanding perceived effects on learning and wellbeing. Specifically, the interview guide included questions about professional development, work-life balance and potential health and psychological changes. Therefore, the exploration produced two analyses: the first focused on learning and developing professional identity, already published (16), and the second presented here focuses on wellbeing.

### 2.4. Data analysis

We followed an iterative data collection and analysis process. ZS and JK read the transcripts and developed a codebook together by open coding. Specifically, constant comparative analysis was used to compare the data across categories to identify and refine thematic patterns, both examining data across transcripts and collecting new data to enrich the evolving analysis (16). During this process, we reviewed data excerpts translated into English to “check” (25) interpretations and to identify opportunities to generate a deeper understanding. The research team used Quirkos Version 2.3.1 to manage the data.

### 2.5. Research team and reflexivity

Data were co-constructed between participants and researchers, meaning that, as researchers, we brought our knowledge, beliefs, and experiences into the research. The research team consisted of various members with different backgrounds and experiences, as well as expertise in medical education, qualitative research, and patient care. BB, RS, AP, AM, RB, CN, JK, and KL were all healthcare professionals involved in different aspects of medical education and patient care. The team members regularly supervised and taught medical students, emphasizing their understanding of the medical education process. Our individual pandemic-related situations [working as a surgical educator, while pregnant during a pandemic (JK), home-schooling children amidst work-related responsibilities (AM, KAL), leading the TTC and working in the ED of a tertiary, academic hospital (RB, CN), disruptions to traditional postgraduate (RS, AP) and undergraduate medical education (ZS, BB)] colored both the questions we asked during interviews and how we interpreted the data.

## 3. Results

Twelve female and nine male participants between the ages of 20 and 26 and in years 2–6 of medical school participated in this study. Most participants described initial fears about the impact of the pandemic on others, with some participants expressing concerns about becoming infected themselves. The work on the frontlines was both physically and mentally demanding but switching to online learning and canceling several exams lifted some of this stress. Interestingly, most medical students perceived the experiences on the frontlines as positive, and COVID-19-related disruptions were perceived by students as a relief from typical medical student agendas and responsibilities, even for those working on the frontlines: “*Everything was cancelled. Suddenly, I did not have volleyball training twice a week or a match. I had a lot more time for myself. and I have to say that it has improved my mental health” (P15).* For participants, working on the COVID-19 frontlines was a coping strategy for “*having a daily structure” (P5), “being kept busy” (P20), “being able to leave the house” (P2), and “staying socially engaged” (P1).* Below, we illustrate these findings with representative quotes from the participants. The following headings provide an overview of the key aspects examined in our study and each heading represents a distinct area of investigation that contributes to a comprehensive understanding of the challenges faced by medical students.

### 3.1. Perceptions of the impact of COVID-19

The initial reactions of medical students to the pandemic ranged from “*it’s just another flu*” (P16) to “*an apocalyptic scenario*” (P18), illustrating the uncertainty everyone was managing early on. Most participants did not realize the impact and severity of COVID-19 during the early stages of the pandemic: “*I just observed the development of the coronavirus and thought to myself: ‘It is an infectious disease like the flu, which is just there, but does not do anything hazardous*” (P1). Others felt concerned or even frightened about the new virus, describing: “*At the beginning, I took it a lot more seriously and had a bit of this doomsday mood, so I thought: ‘Oh my God, we all have to die!*”’ (P18). While most participants held either of these general views on the pandemic, they all reported distinct instances when they realized that the virus was serious, and that the pandemic would indeed impact their daily lives.

The pandemic brought rapid and unexpected disruptions that had a unique impact on the mental health and stress of medical students. They faced challenges as both members of the general public and as students. Social lives changed drastically, and educational experiences were greatly affected, with medical universities closing, exams being cancelled, and in-person lectures shifting online. These disruptions were particularly disheartening for senior medical students, making graduation feel disappointing and anti-climactic.

### 3.2. Feeling safe, but fearful of infection

For some participants, their wellbeing changed over time as knowledge about the virus increased. The possibility of infection was a recurrent issue for the medical students, however. Within their role in the TTC, they learned how to protect themselves through the appropriate use of personal protective equipment (PPE):

*“I liked that we were protected to avoid getting infected with the virus; they provided good masks, plus glasses and all sorts of things. I think hygiene was pushed a lot in the days at Preacher’s Church. The hospital hygiene staff came every day to a point where it was almost annoying” (P9).*

However, not all participants felt safe using PPE. Some students persevered with working at Preacher’s Church despite their anxiety that they might get infected: *“While taking the swab, I just had a lot of trouble with these masks, they were never airtight on me. And then the air still came out to the right and left, even if I tied knots. my feeling was just: ‘I do not think I’m well protected now”’ (P7).* Another participant explained, *“When I made swabs the whole time, I sometimes felt that I could easily get infected, even if I took all protective measures” (P16).*

Most students did not fear catching COVID-19 during their time at the TTC, however: *“Fear would be exaggerated, but I’ve already had a little respect. And then I just thought to myself: ‘No matter where you go, the risk of getting infected is the same or even higher than in Preacher’s Church” (P5).* Instead, most medical students accepted the risk of catching the virus, viewing it as a tradeoff for having the opportunity to work, learn, and serve the community by volunteering at the TTC: *“At the beginning, I had this attitude that I absolutely have to help, that I’m young and if I would catch the virus, it wouldn’t be so bad*…*” (P18).* As one participant outlined*, “There was no reason for me not to work at the Church, even if I put myself in danger. I do it for the society” (P15).* Another student highlighted: “… *it is not just about me and my health but also about the welfare of everyone and those who couldn’t do it for themselves.” (P4).*

### 3.3. Mental health, stress factors, and coping strategies

While medical students faced a mix of stressors during the pandemic, they also experienced relief as certain educational responsibilities were canceled or shifted online, allowing them to prioritize their wellbeing. Notably, exams were conducted virtually and had a more formative nature rather than being high-stakes assessments. One student reported, “*I think that took the stress off a bit. I studied anyway, but never with the intensity that I usually do.*” (P13). Another participant confirmed: “*The pressure from the exam was pretty much off for me because the exam was only formative, and therefore, I had less stress during my studies*” (P5).

One of the biggest challenges overall was maintaining a daily life structure. As one participant explained:

*“For me, the biggest challenge was somehow still finding a daily routine. Well, at first, I did not think it was that bad to always be at home; it was also a relief somehow because otherwise, there is always a lot going on. I thought to myself that I could simply cancel all appointments without a bad conscience*… *and then it was just more and more difficult to maintain a daily routine*…” (P15).

Despite potential risks and added stresses, working permitted social connection to others. At the beginning of the pandemic, students felt isolated, recognizing: “*I’ve had moments once or twice when I felt alone and thought I could not meet anyone and do anything anyway, and the whole situation was still taking too long*” (P1). Therefore, for many medical students, work was a unique opportunity for socialization during the lockdown: *“Otherwise, you were never allowed to see people. The Preacher’s Church [*…*] brought normality into everyday life.”* (P8). The frontlines were also a place to build new relationships and to network: “*I made a couple of good friends from the 6th annual course with whom I even meet for riding bikes*” (P1). Participants mentioned positive social interactions and collaborations with other medical students and the supervising physicians, nurses, and military service members working at the TTC. One student reflected on the interactions with various people: “*You came into contact with others quite a lot. We had lunch together, also with the soldiers, I thought that was cool too*… *a completely new atmosphere that came into the Preachers Church*” (P12). Another participant summarized their experiences as “*fun, above all you finally saw people again, also from different annual courses, and I found it interesting with the soldiers because they were people with whom I would otherwise never have had contact*” (P1).

Generally, participants reported being positively affected by working on the pandemic frontlines, interpreting it as a coping mechanism that allowed them to escape the confines of their lockdown reality. One participant reported, “*Psychologically, I felt some sort of relief because if you can do something in such a crisis, it takes away the feeling of powerlessness*” (P18). Doing something with purpose and structure motivated some participants to join the frontlines: “*You were able to take a lot of motivation out of it because you knew that you would do something useful with your time. Somehow it often did not feel like working at all*” (P11).

## 4. Discussion

The current study provides some insights into how medical students managed their health and wellbeing and which coping strategies they used to maintain these phenomena within their dual roles as learners and healthcare providers. Most participants had experienced both sides of the pandemic. On the one side, participants’ fear and uncertainty caused by the novel coronavirus and lockdowns aligned with the pandemic-related experiences of medical students in other contexts (16). Participants’ experiences also resonate with the four types of pandemic-related fears described by Schimmenti et al. (26). The first is the fear of or for the body, in the case of medical students working on the frontline and getting infected due to inappropriate use of PPE. Second, fear for significant others, meaning that the medical students feared a family member getting sick or worse, catching COVID-19 due to their work or infecting close friends or family members. Third is the fear of not knowing or knowing, such as the stress of uncertainty and unpredictability. Finally, there was fear of taking action or inaction, where medical students reported feeling obligated to help during a crisis, but with reluctance.

**FIGURE 1:**
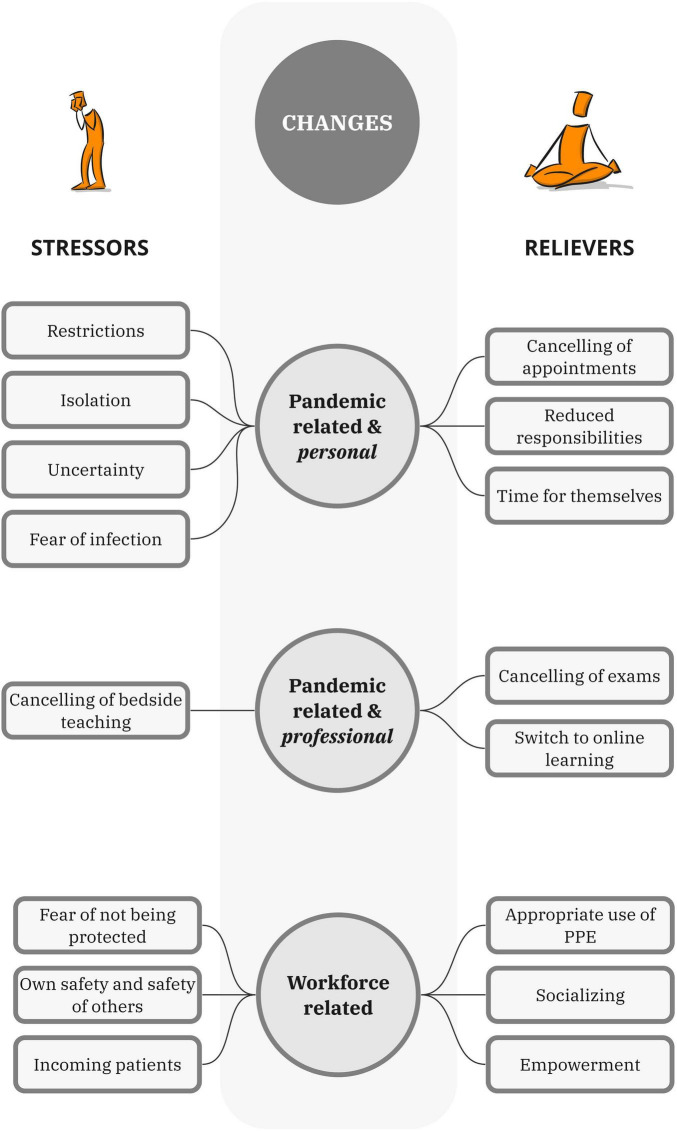
Factors contributing to amplification and relief of pandemic and workforce-related stress.

Despite the stressors participants faced, they were also relieved and grateful for the opportunity to learn and work on the pandemic frontlines, describing their incorporation into the workforce as a positive experience that allowed them to feel useful while also serving their health and wellbeing needs. Indeed, in contrast to earlier quantitative studies reporting a decline in the mental health of medical students during the pandemic (8, 9), our participants’ accounts suggest that certain disruptions and advancements in medical education during the early stages of the pandemic may have had a stabilizing and positive impact on the wellbeing of some students. These observations align with the findings of Luong et al. (27), who reported that having more time for self-care was perceived to facilitate wellbeing despite the chaos and trauma of a global pandemic. For participants in our study, we speculate that their active involvement in a unique learning environment was a significant factor affecting their wellbeing during the pandemic. It served a purpose in permitting the students to socialize at a point in the pandemic when most of the world was locked down. Based on our findings, we synthesized a conceptual model (Figure 1) showing that medical students’ health and wellbeing depend on various work- and pandemic-related factors.

Having more time for self-care and engaging in altruistic opportunities are known to mitigate burnout (28, 29). Moreover, in the medical education context, high stakes assessments are notorious for creating angst amongst learners that can interfere with learning and impair wellbeing (30). It’s little wonder that participants were relieved when traditional assessments were adapted to a lower stakes format early in the pandemic. What is surprising is how the potential benefits of these disruptions and innovations have been overlooked—or perhaps disregarded—amidst a widespread desire to, return to, normal.

Can educational disruptions and innovations like the TTC have value for learner wellbeing beyond the pandemic context? Given that the burnout crisis in medicine is worsening despite decades of efforts to mitigate it, creativity is urgently needed. For instance, although service-learning opportunities are regularly offered across undergraduate medical education (31) they are rarely, if ever, framed as a wellbeing intervention. Despite having selected a profession that focuses on treating and healing others, many medical students lack hands-on experience until the latter part of their training. Participants’ incorporation into frontline service creates special learning environments where students can advance their clinical skills in the midst of a new and previously unfamiliar environment, as demonstrated in our prior research (18).

Those who learn coping skills are significantly less likely to experience feelings of insecurity or depression (32). We found that working on the COVID frontlines helped medical students find their way back to reality again. Furthermore, active involvement in pandemic-related work provided purpose and freedom for medical students, fostering social connections and new relationships. Virtual learning relieved exam-related pressures and allowed flexible study routines. These positive experiences raise the question of expanding virtual platforms and online courses for improved wellbeing and learning outcomes. However, it is important to consider that the overuse of online communication and learning platforms could also be harmful to students’ wellbeing, potentially promoting increased isolation and burnout (33). Together with the deprivation of their home environment, these additional considerations might only help exacerbate the already prevalent mental health issues among medical students (34).

The increased psychological stress experienced by clinicians and medical students can result in adverse consequences. At an individual level, it can lead to mental health issues, physical health challenges, reduced career satisfaction, academic difficulties, and compromised job performance (35, 36). Socio-politically, it strains the healthcare workforce, impacts the quality of care, and increases healthcare costs. Economically, it leads to productivity loss, extended training periods, and higher staff turnover rates, all contributing to financial burdens on healthcare institutions. Addressing stress through support and policy measures is essential for mitigating these far-reaching effects on both individuals and the healthcare system (37).

While engaging medical students in clinical work from early on can be beneficial in returning empowerment by acting as a coping mechanism and improving mental health (38), some potential drawbacks need to be carefully considered and addressed to ensure the wellbeing of students and quality of patient care. These include increased stress and burnout due to the challenges of balancing academic coursework, clinical duties, and personal lives. Early engagement may also result in limited exposure to foundational knowledge and theoretical concepts, potentially compromising students’ understanding and competence. The time and energy required for clinical work may impact students’ academic performance, necessitating careful balance (39).

However, when innovating curricula to enhance trainee wellbeing, it is also crucial to consider the potential impact on faculty wellbeing. While improving trainee wellbeing is important, the added responsibilities and burdens placed on faculty members during the implementation of new initiatives can potentially strain their own wellbeing and work-life balance. To address this, it is essential to provide adequate support, resources, and opportunities for faculty members to ensure that their wellbeing is prioritized. Collaboration and open communication between faculty and administration are key to identifying and mitigating potential challenges and barriers. By taking a comprehensive approach that considers the wellbeing of both trainees and faculty, curricular innovations can be implemented to support the overall wellbeing of the educational community (40).

Medical schools should adopt a holistic curriculum approach that integrates interdisciplinary learning and collaboration to address the physical, emotional, and social wellbeing of students. Longitudinal support programs should be implemented to provide ongoing guidance and resources throughout their training and careers. It is crucial to promote research and evaluation to refine interventions based on feedback from students and educators. Furthermore, medical education workplaces should foster discussions supporting work-life integration and incorporate lessons from the pandemic to enhance the learning environment and student support systems. Mindfulness and self-care workshops can empower students to prioritize their wellbeing and effectively cope with stressors in their professional lives (27). Reflective practices and self-assessment exercises within the curriculum can help students regularly assess their wellbeing and take proactive steps to maintain their mental health (28). Establishing wellbeing committees that facilitate open communication between administration, faculty, and students can also alleviate anxiety and ensure a supportive environment for seeking assistance and addressing discomfort (12).

### 4.1. Limitations

Our study has several limitations that should be considered when interpreting the findings. Firstly, it’s important to note that our research was conducted during the first wave of the COVID-19 pandemic. Consequently, the experiences and perceptions of medical students may have evolved over time, and the applicability of our findings to later phases of the pandemic may be limited. Additionally, limitations of our study include that it exclusively presents the perspectives of medical students who willingly participated in interviews regarding their experiences within a new learning environment at the TTC. It is important to acknowledge that these findings may not encompass the views of all medical students who were part of the TTC.

Furthermore, all interviews were conducted by ZS, a fellow medical student, with the intention of fostering an informal and open dialog among peers on this subject. Nevertheless, we recognize that certain participants might have chosen not to address sensitive topics during this unprecedented epidemic period, potentially impacting the comprehensiveness of our findings. Our study also took place in a Western-country context where medical students had the opportunity to work voluntarily on the COVID-19 frontlines. This circumstance may constrain the transferability of our findings to medical students in settings where such experiences were less common. We acknowledge that medical education and the pandemic’s impact on students can vary significantly across different regions and cultures. While our highly contextualized student experiences closely resemble those reported in other studies, variations in healthcare systems and pandemic responses across different regions could result in different experiences among medical students (27). Collecting data from another country or other regions with different restrictions or burdens of the pandemic may have described different lived experiences (6, 20, 21). Lastly, while our study suggests the importance of tailored support systems and a holistic curriculum approach for medical students, we did not provide specific recommendations or strategies for implementing these changes in medical education. These limitations should be taken into account when interpreting our findings and considering their applicability in different contexts and phases of the pandemic.

## 5. Conclusion

The COVID-19 pandemic has been disruptive to medical students. This study highlights the experiences of medical students on the COVID-19 frontline regarding relief, stress, and mental health. Despite the challenges they faced, participants expressed relief and gratitude for the opportunity to work on the frontline, describing it as a positive experience that fulfilled their sense of purpose and addressed their health and wellbeing needs. The findings emphasize the need for tailored support systems, including longitudinal programs, mindfulness workshops, and open communication. Addressing these challenges is crucial for improving the wellbeing of medical students and enhancing healthcare delivery during crises.

## Data availability statement

The raw data supporting the conclusions of this article will be made available by the authors, without undue reservation.

## Ethics statement

The studies involving humans were approved by the Ethics Commission of Northwest and Central Switzerland (EKNZ, Req-2021-00518). The studies were conducted in accordance with the local legislation and institutional requirements. The participants provided their written informed consent to participate in this study.

## Author contributions

JK and ZS: conceptualization, methodology, software, quirkos 2.3.1, and data curation. JK and AP: validation and writing—review and editing. JK, ZS, RS, AP, BB, and KL: formal analysis. ZS: investigation. CN and RB: resources. JK, AP, RS, and ZS: writing—original draft preparation. JK, AM, and KL: supervision. All authors have visualization, read, and agreed to the published version of the manuscript.
