# Supplementary material for: Medical students on the COVID-19 frontline: a qualitative investigation of experiences of relief, stress, and mental health
Source: Front Med (Lausanne). 2023 Nov 9;10:1249618. doi: 10.3389/fmed.2023.1249618 (PMC10666052; doi:10.3389/fmed.2023.1249618)
Supplement: Supplementary file 1 [file Data_Sheet_1.docx]

**Interview guide Students Preacher’s Church**

Research questions:

WHY did medical students apply to the COVID-19 task force?

HOW do medical students perceive working on a pandemic frontline?

WHAT experiences did they gain by working in the COVID-19 task force?

HOW are these experiences going to influence their current and future professional identity?

Demographics: male, female, age, year of study.

Date of interview (circumstances of lockdown/RE lockdown).

Circumstances

**Field notes Interview X_06/07/20:**

- Date, Name, Gender:

- Environment/Situation:

- Relationship:

- Feeling:

- How did it shape the data:

- "Eye-Catching":

- Previous jobs:

*Starting questions (Easing-in questions):*

1. Please describe your previous medical (professional) experience
2. What made you want to study medicine?
3. What did a typical day look like for you before COVID-19? (Responsibilities, at home, at study, ...)

*Intermediate part:*

1. What did a typical day look like for you during the COVID-19 high phase/lockdowns?
2. What does a day look like for you this week?
3. Explain how exactly your training/study situation has changed as a result of COVID-19.
4. Do you remember the moment when you first realized that your previous life was going to change?
5. How has your perspective on the situation changed from the beginning to now?
6. What has been the biggest challenge for you since the COVID-19 pandemic began?

- Personally?
- Professionally?
- What was easy to change?

1. What made you sign up for the frontline?
2. What did you expect/expect from the work?
3. What did you learn at work?
4. Which expectations were not met and why?
5. How did those around you react when you started working on the Corona Task Force?
6. Were you afraid of infecting yourself with COVID-19?

- Why yes/no?
- Did you feel adequately protected?

1. How was your first day?
2. What did a typical working day look like?
3. What did an extraordinary working day look like?
4. Was war für dich ein prägendes Erlebnis mit Patienten?
   🡪Warum? Was hast du mitgenommen?
5. What was a formative experience for you with patients?

Why? What did you take with you?

1. What was your most traumatic experience?

If none: What situation made you brood?

1. What did working at the Task Force do for your studies?
2. What has working at the Task Force taught you about life?
3. Would you do it again? Why yes/no?
4. How has your work-life balance evolved under COVID-19?
5. Have you noticed any changes in your physical or mental health?
6. What was your coping strategy?

- Rejection
- Information gathering vs. rejection
- Sharing with family/friends
- Sports/hobbies new or rediscovered (alone in the woods)
- Social media

*Ending questions:*

1. What positive side effects could you see during the time shaped by Corona?

- in private life
- in professional life/studies
- in society in general

1. What can we as a society learn from the pandemic?
2. What do you take away from this situation? Will this experience change/influence your normal life? In what way?
3. If you knew then what you know now, what tips would you have given yourself??
4. How have priorities and values changed for you?
5. Before we end the interview, do you have anything to add?
6. Would you be available for a possible follow-up interview?
